# Epigenetic inactivation of *ST6GAL1* in human bladder cancer

**DOI:** 10.1186/1471-2407-14-901

**Published:** 2014-12-02

**Authors:** Pia Antony, Michael Rose, Axel Heidenreich, Ruth Knüchel, Nadine T Gaisa, Edgar Dahl

**Affiliations:** Molecular Oncology Group, Institute of Pathology, RWTH Aachen University, Pauwelsstrasse 30, 52074 Aachen, Germany; Department of Urology, University Hospital Aachen, Pauwelsstrasse 30, 52074 Aachen, Germany

**Keywords:** ST6GAL1, Bladder cancer, DNA methylation, Tumor suppressor

## Abstract

**Background:**

Posttranslational protein modifications are known to modulate key biological processes like proliferation and apoptosis. Accumulating evidence shows that ST6GAL1, an enzyme that catalyzes the transfer of sialic acid onto galactose-containing substrates, is aberrantly expressed in various cancers and may affect cell motility and invasion. This is the first study to describe ST6GAL1 expression and regulation in human bladder cancer.

**Methods:**

*ST6GAL1* mRNA expression levels in human cell lines (UROtsa, RT4, RT112 and J82) and tissue samples (n = 15 normal urothelium (NU), n = 13 papillary non-invasive tumors (pTa), n = 12 carcinoma in situ (CIS), n = 26 muscle invasive tumors (pT2-4)) were assessed using real-time PCR. In addition, ST6GAL1 protein expression was evaluated using immunohistochemistry. Promoter methylation analysis was performed using methylation-specific PCR (MSP) in cell lines (n = 4) and patient samples (n = 23 NU, n = 12 CIS, n = 29 pTa, n = 41 pT2-4). Epigenetic *ST6GAL1* gene silencing was confirmed by *in vitro* demethylation of bladder cell lines. Data were validated by analysis of an independent bladder tumor data set (n = 184) based on *The Cancer Genome Atlas* (TCGA) portal.

**Results:**

Semi-quantitative *ST6GAL1* real-time PCR expression analysis showed two distinct trends: In muscle-invasive tumors *ST6GAL1* expression was downregulation by 2.7-fold, while papillary non-invasive tumors showed an increased *ST6GAL1* mRNA expression compared to normal urothelium. *ST6GAL1* loss in muscle-invasive tumors was associated with increasing invasiveness. On the protein level, 69.2% (n = 45/65) of all tumors showed a weak ST6GAL1 protein staining (IRS ≤ 4) while 25.6% (16/65) exhibited a complete loss (IRS = 0) of ST6GAL1 protein. Tumor-specific DNA methylation of the *ST6GAL1* promoter region was frequently found in pT2-4 tumors (53.6% (22/41)), whereas only 13.8% (4/29) of pTa tumors showed *ST6GAL1* promoter methylation*.* Normal urothelium remained unmethylated. Importantly, we significantly revealed an inverse correlation between *ST6GAL1* mRNA expression and *ST6GAL1* promoter merthylation in primary bladder cancer. These findings were clearly verified by the TCGA public data set and *in vitro* demethylation assays functionally confirmed *ST6GAL1* promoter methylation as a potential regulatory factor for *ST6GAL1* gene silencing.

**Conclusions:**

Our study characterizes for the first time ST6GAL1 expression loss caused by aberrant *ST6GAL1* promoter methylation potentially indicating a tumor suppressive role in bladder carcinogenesis.

**Electronic supplementary material:**

The online version of this article (doi:10.1186/1471-2407-14-901) contains supplementary material, which is available to authorized users.

## Background

Urinary bladder cancer currently represents the 5^th^ most common cancer type in the industrialized nations [1]. Clinically, bladder cancer poses a unique clinical challenge, consisting of a heterogeneous group with either recurrent, but relatively benign course or progressive oncological course [2]. In 90% of cases, tumors arise from superficial cell layers in the urogenital tract known as urothelial cells (formerly transitional cells) [3]. Here, two major growth forms, papillary non-invasive and flat-invasive, have been identified, underlying two separate molecular pathways characterized by distinct mutations [4]. Low-grade tumors, which display a papillary growth form and are mostly superficial (Ta low grade urothelial carcinoma (UC)), constitute the largest group at diagnosis, and are characterized by their high recurrence rate. The second group is formed by high-grade tumors and includes carcinoma in situ (CIS), a flat high-grade lesion, which in 60–80% of cases progresses to invasive bladder cancer within 5 years [3]. Low stage/grade tumors are often characterized by mutations in *fibroblast growth factor receptor 3 (FGFR3)* [5, 6] and *rat sarcoma (RAS)* genes [5, 7], while flat carcinoma in situ lesions frequently show mutations in *TP53* in addition to a loss of heterozygosity of the retinoblastoma (RB) gene [3, 5, 8, 9]. As such high grade tumors progress to a muscle invasive stage, an ever increasing degree of DNA hypermethylation is observed [10]. Current research aims to further elucidate the changes defining these divergent growth forms given their different clinical impact and therapeutic needs.

Post-translational modifications are known to influence protein characteristics such as protein folding and stability, thus modulating biological processes like cell growth and migration [11]. As a result, altered glycosylation of proteins, such as cell surface glycoproteins and glycolipids, is a common and frequent feature during carcinogenesis, due to impaired activity of glycosyltransferases [12]. The *ST6GAL1* gene encodes a type II membrane protein (beta-galactosamide alpha-2,6-sialyltransferase 1, ST6GAL1) that catalyzes the transfer of sialic acid from cytidine-monophosphate (CMP)-sialic acid onto galactose-containing substrates [12–14]. Previous studies have shown that ST6GAL1 functions as a critical regulator of cell survival in several cell death pathways [14, 15]. For example, its sialylation of the Fas death receptor hinders internalization of Fas after activation, thereby reducing apoptotic signaling [14]. Similarly, ST6GAL1 promotes cell surface retention of the tumor necrosis factor receptor 1 (TNFR1) and the CD45 receptor [14, 15]. Furthermore *in vitro* studies have shown that ST6GAL1 upregulation promotes cell migration and invasion through its interaction with the B1 integrin receptor [16–19], while animal models of colon cancer implicate ST6GAL1 in tumor invasiveness [20]. While these studies clearly underline the oncogenic potential of ST6GAL1, seemingly contradictory evidence has emerged suggesting it may also have tumor suppressive qualities [21]. For example, recent studies clearly showed that a downregulation of ST6GAL1 activity in colorectal carcinoma cell lines facilitated cell proliferation and tumor growth [21].

However, the impact of ST6GAL1 in bladder cancer remains unclear to date. We found *ST6GAL1* downregulated in bladder cancer samples in a previous metg001A Affymetrix® GeneChip study reported by Wild et al. [22]. Therefore, the current study seeks to further elucidate ST6GAL1 expression and its regulation in order to determine the potential impact of the glycosyltransferase ST6GAL1 on bladder cancer development.

## Methods

### Urothelial cell lines

The human SV40-transfected urothelial cell line UROtsa, initially generated from normal ureter tissue, and the papillary-invasive urinary bladder cancer cell lines RT4 and RT112, as well as the invasive bladder cancer cell line J82 from ATCC (*American Type Culture Collection,* Manassas, Virginia*,* USA) were cultivated according to the manufacturer’s instructions.

### Patient materials

Formalin fixed paraffin embedded (FFPE) tumorous bladder tissue samples analyzed in this study were obtained from our pathology archive and the “whole bladder sampling project (bladder mapping)” integrated in the tumor bank of the Euregional comprehensive Cancer Center Aachen (ECCA), now part of the RWTH centralized biomaterial bank (RWTH cBMB; http://www.cbmb.rwth-aachen.de). All cBMB patients gave written informed consent for retention and analysis of their tissue for research purposes according to local Institutional Review Board (IRB)-approved protocols (approval no. EK-206/09, EK-122/04 and EK 173/06) of the medical faculty of the RWTH Aachen University. For cohort characteristics of all analyzed samples see Table 1. Additionally, 15 normal tissue samples of patients were used. In order to prevent contamination from surrounding tissue, urothelium or tumor tissue was isolated from multiple tissue sections via manual micro-dissection under a stereo microscope, respectively.

**Table 1:** **Clinico-pathological parameters of all 109 bladder cancer specimens analyzed in this study (RT-PCR/MSP/immunohistochemistry)**

	Categorization	n ^a^analyzable	%
***Parameter:***			
Age at diagnosis:	Median: 69 years		
	(range 26–94)		
	≤69 years	55	50.5
	>69 years	54	49.5
Gender			
	Female	29	26.6
	Male	80	73.4
Tumor subtype			
	Carcinoma *in situ*	13	11.9
	Papillary non-invasive	17	15.6
	Invasive	79	72.5
Histological tumor grade^b^			
	G1	14	12.8
	G2	9	8.3
	G3	80	73.4
	unknown	6	5.5
Histological tumor grade^c^			
	Low grade	15	13.8
	High grade	94	86.2
Tumor stage^c^			
	pTx	1	0.9
	pTis	13	11.9
	pTa	16	14.7
	pT1	12	11.0
	pT2	25	22.9
	pT3	31	28.4
	pT4	11	10.1
Concomitant CIS			
	Negative	26	23.9
	Positive	52	47.7
	Unknown	31	28.4
Lymph node status			
	Negative	41	37.6
	Positive	18	16.5
	Unknown	50	45.9

^a^Only patients with primary bladder cancer were included; ^b^according to WHO 1973 classification; ^c^according to WHO 2004 classification.

### DNA and RNA extraction

DNA extraction was achieved using the QiAmp-DNA-Mini-Kit (Qiagen, Hilden, Germany), according to the manufacturer’s instructions. RNA was isolated using the TRIzol approach specified by the manufacturers (Invitrogen, Carlsbad/CA, USA).

### Reverse Transcription PCR (RT-PCR)

A total of 1 μg RNA was translated into cDNA using the Promega-Reverse-Transcription-System (Promega, Madison/WI, USA), according to the manufacturer’s instructions In order to maximize the cDNA yield, Oligo-dTs and hexameric random primers (pdN(6)) were mixed in a ratio of 1:2. A total of 1 μl cDNA (20 ng) was used for the PCR reaction.

### Semi-quantitative real-time PCR

Real-time PCRs were performed using the iCycler system iQ5 (Bio-Rad Laboratories, Munich) with an intron bridging primer, according to the manufacturer’s instructions. The ubiquitous housekeeping gene *glyeradehyde 3-phosphate dehydrogenase (GAPDH)* was used as a reference gene. For analysis, a cut off value for *GAPDH* was assigned at a value of 30. The sequences of the applied primers were as follows: *GAPDH* 5′-*sense*: 5′-GAA GGT GAA GGT CGG AGT CA-3′; 3′-*antisense*: 5′-AAT GAA GGG CTC ATT GAT GG-3′ with a product size of 108bp. *ST6GAL1* 5′-*sense*: 5′-TGT CTA GAA AAG AAG GTG GAG ACA T-3′; 3′-*antisense*: 5′-AGG GTC CTG TTG GCA TTC TC-3′ with a product size of 89 bp. The annealing temperature for both primer sets was 60°C. The relative mRNA-quantification was analyzed using comparative CT methods in comparison to *GAPDH*-expression.

### Bisulfite-modification and methylation-specific PCR (MSP)

Bisulfite conversion of 1 μg of all genomic DNA was achieved using the EZ-DNA-Methylation-Kit (Zymo Research, Orange/CA, USA) and the precipitate was eluted in 20 μl of Tris-Ethylenediaminetetraacetic acid (EDTA)-buffer. Thereafter, 1 μl was amplified in a methylation specific PCR using an optimized PCR buffer. MSP-Primers, specific for the unmethylated *ST6GAL1*-promotor sequence, were used and read as follows: 5′-GAA GAC GTT TGG GGT ATT GTT CGG C-3′ (M *sense*) and 5′-TAC ACT CTC GAC CGC GAA AAC TAC G-3′ (M *antisense*); 5′-GGG AAG ATG TTT GGG GTA TTG TTT GGT G-3′ (U *sense*) and 5′-TCA CTC ACT ACA CTC TCA ACC GCA AAA ACT ACA-3′ (U *antisense*). The reaction consisted of 400 nM of the specific primer pairs and 1.25 mM of individual dNTPs. The PCR was completed using the hotstart-PCR method, where 1.25 units of *Taq*-DNA-Polymerase (Roche Diagnostics, Mannheim, Germany) were given to the mixture when the reaction reached a temperature of 80°C. The PCR conditions were: 95°C for 5 min, followed by 35 cycles of the following sequence: 95°C for 30 s, 56°C for 30 s, 72°C for 30 s and 72°C for 5 min. The amplified PCR product was then run on a 3% low-range ultra-agarose gel (Bio-Rad Laboratories, Hercules/CA, Germany) with ethidium bromide and then visualized using ultraviolet light.

### *In vitro*demethylation of genomic DNA

Bladder cell lines were seeded in a 6 well dish with a concentration of 3 × 10^4^ cells/cm. A demethylating substance, 5-aza-2′-deoxycytidine (DAC) in a concentration of 1 μM (Sigma-Aldrich, Deisenheim, Germany), was added with fresh medium on day 1, 2 und 3. In addition, 300 nM of the histone deacetylase inhibitor trichostatin A (TSA, Sigma-Aldrich) was added on day 3. The cells were cultivated in fresh medium after every treatment and harvested on the fourth day for RNA extraction.

### ST6GAL1 immunohistochemistry

2 μm slides of formalin fixed paraffin embedded (FFPE) bladder tissues were stained with ST6GAL1 monoclonal antibody LN1 clone with 1:100 dilution (MAB6959, Abnova, Walnut, CA, USA). Heat induced antigen retrieval in pH 9.0 EDTA buffer was performed and samples were blocked with peroxide blocking solution from DAKO (DAKO, Hamburg, Germany). DAKO K5007 kit was used as a detection method, according to the manufacturer’s instructions. For visualization *3,3′-*diaminobenzidine (DAB) and haematoxylin counter-stain was used. Staining was evaluated according to an adapted semi quantitative scoring system by Remmele and Stegner [23].

### Validation of *ST6GAL1*expression and promoter hypermethylation in an independent set of bladder tumors

In order to independently assess *ST6GAL1* mRNA expression and DNA methylation we used public data from both primary invasive [24] and papillary bladder cancer tissues from “The Cancer Genome Atlas” (TCGA) data portal (https://tcga-data.nci.nih.gov). These comprise data of overall n = 184 patients from two independent platforms: Illumina Infinium DNA methylation chip (HumanMethylation 450) and Illumina HiSeq gene expression. The data can be explored through the cBio Cancer Genomics Portal (http://cbioportal.org). For cohort characteristics of analyzed TCGA samples in this current study see Additional file 1: Table S1.

### Statistical data analysis

All statistical analysis was done using SPSS 17.0 (SPSS Software GmbH, Munich). Results were considered to be statistically significant given a p value of <0.05. All statistical tests were performed 2 sided. In order to compare two groups, the non-parametric Mann–Whitney U-test was implemented. In case of more than two generated groups the Kruskal-Wallis test and Dunn’s multiple comparison test was used. Any correlation to clinical pathological factors and molecular parameters were analyzed using a descriptive Fisher’s exact-test. Correlation between *ST6GAL1* expression (TCGA Illumina HiSeq platform) and *ST6GAL1* methylation data (TCGA HM450 platform) was performed by calculating a *Spearman* correlation coefficient.

## Results

### *ST6GAL1*mRNA is differently expressed in human bladder cancer

Previously, Wild and colleagues described novel candidate genes differentially expressed in human bladder cancer [22]. Based on that, we re-analyzed the DNA microarray data set by using *in silico* database mining procedures identifying novel candidates with differential expression in the course of bladder cancer progression (data not shown). As such we frequently detected *ST6GAL1* expression loss by 2.6-fold in pTa and by 3.4-fold in pT1/pT2 bladder tumors. Therefore we aimed to study for the first time ST6GAL1 expression and regulation in bladder cancer.

**Figure 1:**
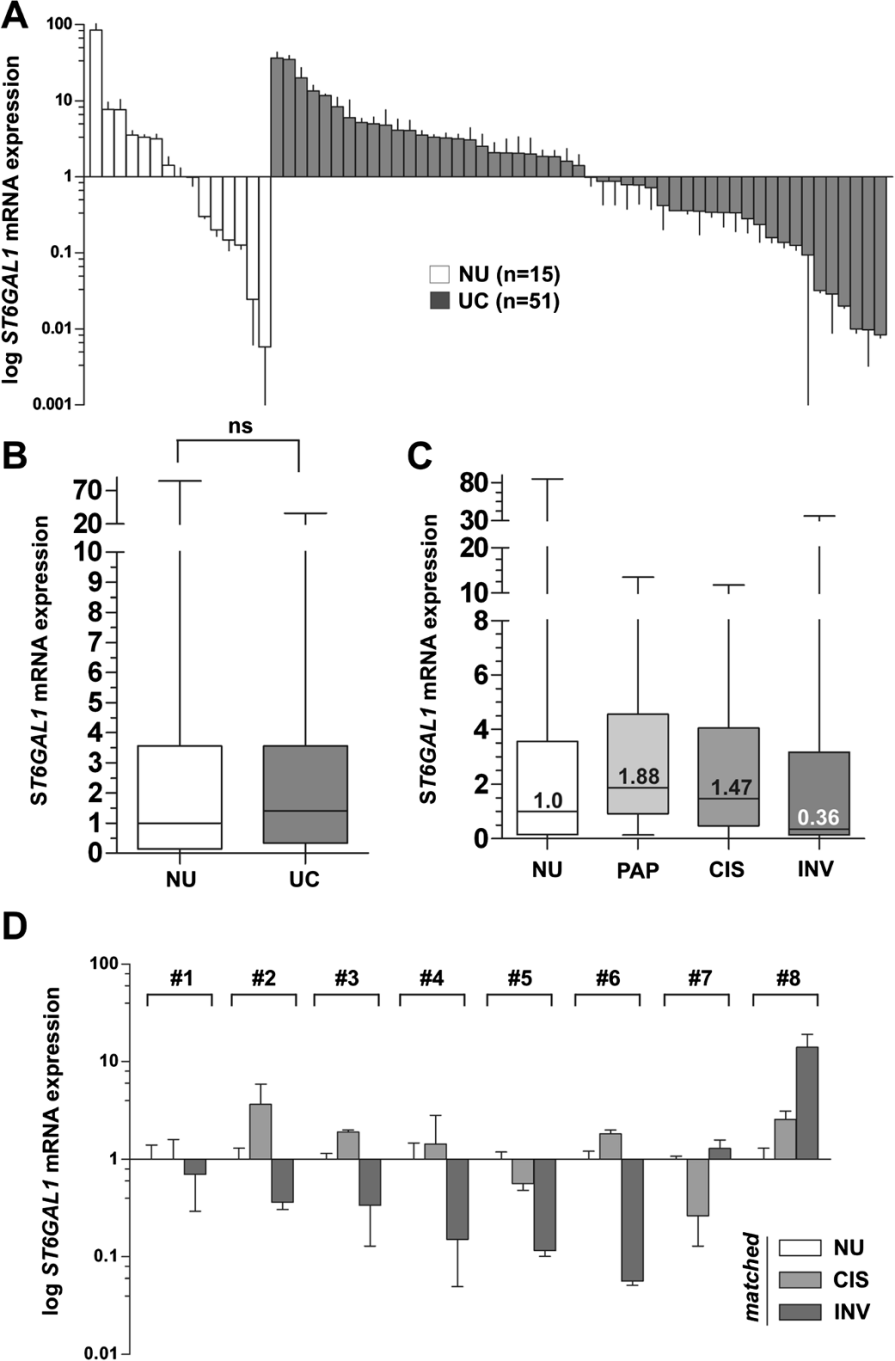
***ST6GAL1*** **mRNA expression analyses in human bladder cancer. (A)** Real-time PCR based *ST6GAL1* mRNA expression analyses of 51 tumor samples (UC) compared to normal urothelium (NU) samples (n = 15). The median expression level of NU was set to 1. Vertical lines: ± standard error of margin (s.e.m.). **(B)** Box plot showing median *ST6GAL1* mRNA expression in normal urothelium compared to all analyzed urothelial cancer samples. *Horizontal lines*: grouped medians. *Boxes*: 25–75% quartiles. *Vertical lines*: range, peak and minimum, *ns*: not significant. **(C)** Itemized box plot demonstrating median *ST6GAL1* mRNA expression in normal urothelium (NU), non-invasive papillary tumors (PAP), CIS, and invasive bladder tumors (INV). *Horizontal lines*: grouped medians. *Boxes*: 25–75% quartiles. *Vertical lines*: range, peak and minimum. **(D)** Real-time PCR based *ST6GAL1* mRNA expression analyses of patient triplets with matched normal urothelium, CIS and invasive tumor samples. Matched CIS and solid tumors were normalized to the corresponding NU, respectively. Vertical lines: + s.e.m.

Semi-quantitative *ST6GAL1* real-time PCR expression analysis was performed on 51 bladder cancer tissue samples including flat (n = 12 CIS), papillary non-invasive (n = 13 pTa), and muscle-invasive (n=26 pT2-4) bladder tumor samples (Figure 1A). Detailed cohort statistics can be found in Table 1. Overall, no significant trend was observed when normal urothelium was compared with a mixed tumor cohort (1B). Classification of these samples by tumor subtypes, however showed that *ST6GAL1* mRNA expression tends to be downregulated (fold change, FC: 0.36) in muscle-invasive tumors when compared to normal urothelium (NU) (n = 15). In contrast, *ST6GAL1* mRNA expression in papillary non-invasive tumors was found to be increased (FC: 1.88) (Figure 1C). A tight association between loss of *ST6GAL1* mRNA expression and advanced bladder tumor stages (pT2-pT4; invasive subtype) was significantly underscored by using Fisher’s exact test (Table 2).

**Table 2:** **Clinico-pathological parameters in relation to** ***ST6GAL1*** **mRNA expression**

		***ST6GAL1***mRNA level ^b^				
		***n*** ^***a***^	low	high	P-value ^c^	Spearman**rs**
***Parameter:***						
Age at diagnosis						
	≤69 years	24	10	14	0.977	-0.004
	>69 years	19	8	11		
Gender						
	Female	9	5	4	0.273	0.169
	Male	41	14	27		
Tumor subtype						
	Non-invasive	13	2	11	**0.017**	-0.402
	Invasive	26	15	11		
Histological tumor grade^d^						
	Low grade	11	1	10	0.035	-0.316
	High grade	39	18	21		
Tumor stage^d^						
	pTa-pT1	16	4	12	0.099	-0.313
	pT2-pT4	23	13	10		

^a^Only patients with primary bladder cancer were included; ^b^cut-off: 0.5 in relation to NU expression; ^c^Fisher’s exact test; ^d^according to WHO 2004 classification, significant p-values are marked in bold face.

Facing this heterogeneous expression in bladder tumors, we performed further real-time PCR expression analysis using eight patient-matched specimens including NU, CIS, and invasive tumor tissues from each single patient allowing a precise assessment of *ST6GAL1* mRNA expression in the course of bladder cancer progression (Figure 1D). In five of the analyzed patients, a clear downregulation (<0.5 FC) of *ST6GAL1* mRNA expression was detected in solid tumors, i.e. in invasive stages. Only one patient showed an increase with a FC >2 in *ST6GAL1* gene expression and two patients an insignificant change (Figure 1D), not impairing a pronounced loss of *ST6GAL1* in invasive tumor stages.

### ST6GAL1 protein expression in human bladder cancer

**Figure 2:**
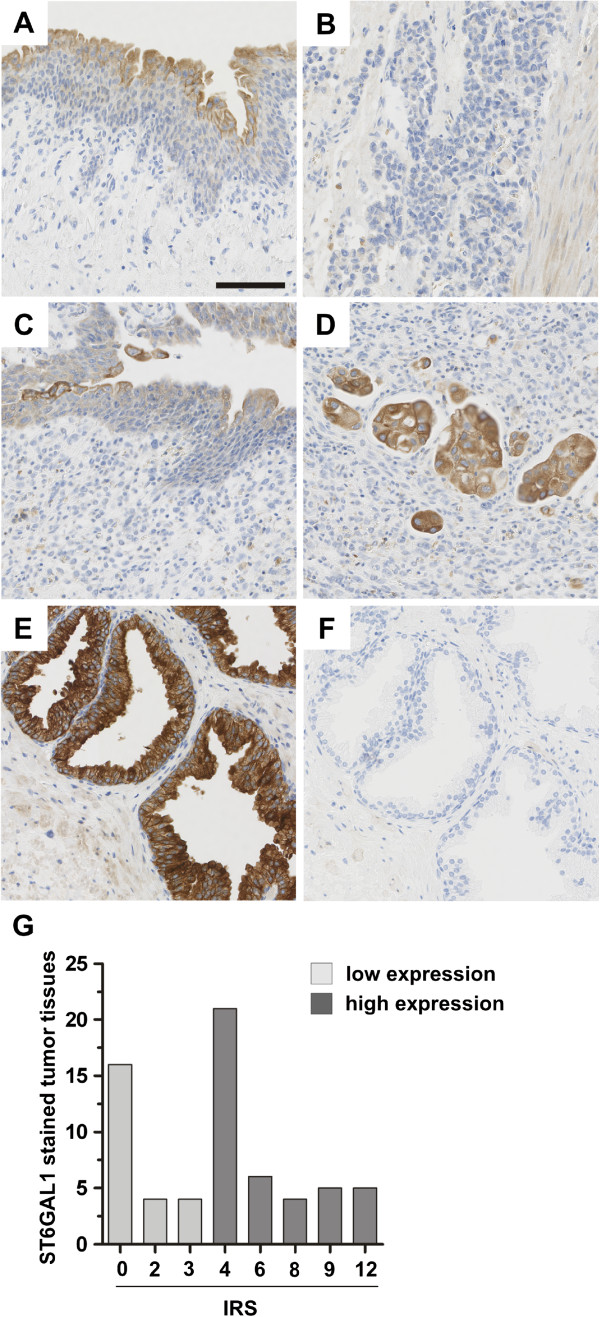
**ST6GAL1 protein expression in human bladder cancer. (A) + (B)** matched samples of patient #1 with **(A)** normal urothelium with accentuated ST6GAL1 protein expression in superficial (“umbrella”) cells and **(B)** complete loss of ST6GAL1 protein in the poorly differentiated invasive bladder cancer. **(C) + (D)** Matched samples of patient #2 with **(C)** normal urothelium with accentuated ST6GAL1 protein expression in superficial (“umbrella”) cells and **(D)** moderate ST6GAL1 protein expression in the invasive bladder cancer (staining intensity 2). **(E)** Positive control: prostate sample with strong ST6GAL1 staining (staining intensity 3). **(F)** Negative control: prostate sample with no staining (staining intensity 0). **(G)** Histogram of ST6GAL1 protein expression (immunoreactive score, IRS) distribution among all tumor samples.

In light of differential *ST6GAL1* mRNA expression in bladder cancer, we performed immunohistochemical ST6GAL1 protein expression in NU as well as in bladder tumor tissues. ST6GAL1 protein staining was quantified according to an adapted immunoreactive score (IRS) developed by Remmele and Stegner [23]. In NU ST6GAL1 was detected cytoplasmatically in all urothelial cell layers, with strongest abundance in superficial (“umbrella”) cells (Figure 2A). In contrast, invasive bladder tumors were characterized by a decreased ST6GAL1 protein staining (Figures 2B-D): 69.2% (n = 45/65) of all analyzed tumors showed an ST6GAL1 protein staining of IRS ≤ 4 and 25.6% (16/65) exhibited an almost complete loss (IRS = 0) of ST6GAL1 protein (Figure 2G). A correlation analysis with clinico-pathological characteristics revealed a significant association (p = 0.017) of ST6GAL1 protein reduction and the existence of lymph node metastasis (Table 3).

**Table 3:** **Clinico-pathological parameters of high grade, invasive bladder tumors in relation to ST6GAL1 protein expression**

		ST6GAL1 IRS ^b^				
		***n*** ^***a***^	low ^(IRS 0–3)^	high ^(IRS 4–12)^	P-value ^c^	Spearman**rs**
***Parameter:***						
Age at diagnosis						
	≤69 years	27	10	17	0.987	0.002
	>69 years	38	14	24		
Gender						
	Female	21	8	13	0.894	0.017
	Male	44	16	28		
Histological tumor grade^d^						
	G2	7	4	3	0.247	0.146
	G3	58	20	38		
Tumor stage^e^						
	pT1-pT2	29	7	22	0.056	-0.238
	pT3-pT4	36	22	19		
Lymph node status						
	Negative	39	11	28	**0.017**	-0.321
	Positive	16	10	6		

^a^Only patients with primary bladder cancer were included; ^b^cut-off: median immunoreactive score (IRS) according to Remmele and Stegner [23]; ^c^Fisher’s exact test; ^d^according to WHO 1973 classification according to WHO 2004 classification; ^e^according to WHO 2004 classification; significant p-values are marked in bold face.

### *ST6GAL1*promoter hypermethylation is associated with ST6GAL1 expression loss in human bladder cell lines

**Figure 3:**
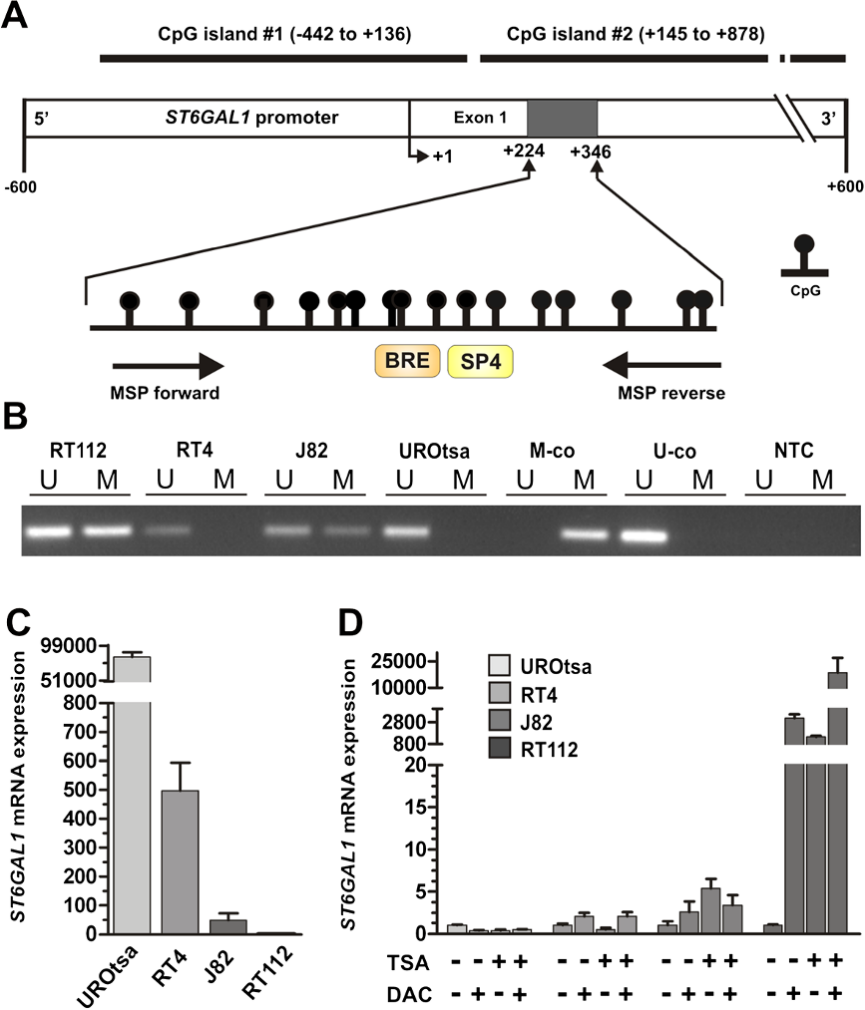
***ST6GAL1*** **promoter methylation in human bladder cell lines correlates with** ***ST6GAL1*** **mRNA expression. (A)** Schematic map of the human *ST6GAL1* promoter region including the relative positions of analyzed CpG dinucleotides using MSP (MSP primer binding sites are indicated by arrows) that is located within the non-coding exon 1 region. Two predicted CpG islands are located between base -442 and base +136 as well as between base +145 and base +878 in relation to the transcription start site (TSS). +1: *ST6GAL1* TSS. Orange and yellow boxes illustrating gene transcription-relevant regulatory elements statistically identified by using the Genomatix data base (http://www.genomatix.de/): BRE: Transcription factor II B (TFIIB) recognition element (score: 1.0, position in relation to TSS: 282–288 bp); SP4: Ubiquitous GC-Box factors SP1/GC recognition element for SP4 TF (score: 0.89, position in relation to TSS: 287–303 bp). **(B)** Representative MSP analysis illustrating the *ST6GAL1* promoter methylation status of human bladder cell lines RT112, RT4, J82 and UROtsa. Band labels with U and M represent an unmethylated and methylated DNA locus. Bisulphite-converted unmethylated, genomic (U-co) and polymethylated, genomic (M-co) DNA were used as positive controls. NTC: non-template control. **(C)** Comparison of *ST6GAL1* mRNA expression of human bladder cell lines showing an unmethylated *ST6GAL1* promoter hypermethylation (UROtsa and RT4) with *ST6GAL1* methylated J82 and RT112 cells. Vertical lines: + s.e.m. **(D)** DNA demethylation of the *ST6GAL1* promoter correlates with *ST6GAL1* re-expression *in vitro.* Real-time PCR of *ST6GAL1* mRNA expression demonstrated a clear *ST6GAL1* re-expression after treatment with both DAC (+) and TSA (+) only in the RT112 bladder cell line. Non-treated cells were set to 1. Error bars: + s.e.m.

Given the observation of ST6GAL1 expression loss in the course of bladder cancer progression, we attempted to decipher the molecular cause for *ST6GAL1* gene silencing. Recently, DNA methylation in promoter regions, a frequent and well-known epigenetic mechanism of gene inactivation during carcinogenesis was shown in breast cancer [25]. Therefore, we assessed whether this epigenetic modification might be responsible for ST6GAL1 downregulation in bladder cancer as well. Analysis of the *ST6GAL1* gene promoter using the genomic DNA information (ENSEMBL contig ENSG00000073849) and the *Methprimer* program [26] identified two CpG-rich islands between genomic positions 186,930,485 and 187,078,553 on chromosome 3 which met the following criteria: DNA region: ≥200bp; Obs/Exp: ≥0.6; %GC: ≥50. The *ST6GAL1* promoter region analyzed by methylation specific PCR (MSP) is located in the non-coding exon 1 next to the transcription start site (TSS) and encodes potential regulatory sequences such as the ubiquitous transcription factor II B (TFIIB) recognition element (BRE: ccgCGCC ) (Figure 3A).

MSP analysis showed indeed distinct DNA methylation in the human bladder cancer cell line J82 and RT112 at the *ST6GAL1* promoter region. Of importance the *ST6GAL1* promoter region of the immortalized bladder cell line UROtsa originally derived from normal urothelium remained unmethylated. Reflecting the variance seen in our tumor population, the RT4 papillary-invasive cell line showed no *ST6GAL1* promoter methylation (Figure 3B). This unmethylated configuration of the *ST6GAL1* promoter correlated with a strong *ST6GAL1* expression in both cell lines UROtsa and RT4, whereas a weak expression in J82 and a nearly complete loss of *ST6GAL1* mRNA expression in RT112 cancer cells was observed (Figure 3C).

*In vitro* demethylation assays of human bladder cell lines (RT112, RT4, J82 and UROtsa) were performed to further underscore the functional association between *ST6GAL1* promoter methylation and gene transcription. Demethylation application led to an upregulation of *ST6GAL1* mRNA expression approximately by 18,000 fold in highly methylated RT112 tumor cells (Figure 3D). *ST6GAL1* mRNA expression in J82 cells was solely marginal re-expressed after DAC and TSA treatment. *ST6GAL1* mRNA in both normal-like UROtsa and RT4 bladder tumor cells harboring an unmethylated *ST6GAL1* promoter served as control and were not further inducible by DAC/TSA (Figure 3D). These data indicate that epigenetic configurations at the *ST6GAL1* promoter region are involved in the regulation of *ST6GAL1* expression.

### *ST6GAL1*promoter methylation is tightly associated with *ST6GAL1*expression loss in primary bladder tumors

**Figure 4:**
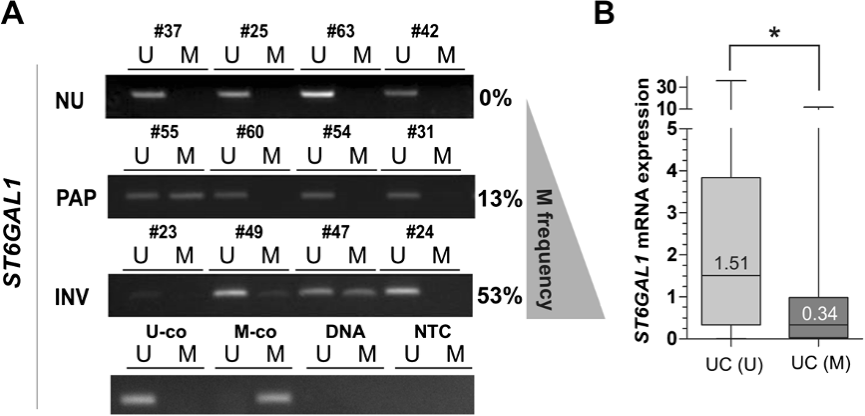
***ST6GAL1*** **promoter hypermethylation in primary human bladder cancer is associated with loss of** ***ST6GAL1*** **mRNA expression. (A)** Representative MSP results of the *ST6GAL1* promoter methylation status in four papillary non-invasive pTa low grade tumors (PAP) as well as four invasive high grade bladder cancer (INV) samples in comparison to four normal tissue specimens (NU). Bands labeled with U and M show unmethylated and methylated DNA, respectively. Percent values reflect methylation frequency (M frequency). Bisulphite-converted unmethylated, genomic (U-co) and polymethylated, genomic (M-co) DNA were used as positive controls. DNA control: genomic, non- bisulphite-converted DNA, NTC: non-template control. **(B)** Box plot illustrating *ST6GAL1* mRNA downregulation according to hypermethylated *ST6GAL1* promoter status in bladder cancer (UC). (U): unmethylated tumors. (M): Methylated tumors. *Horizontal lines*: grouped medians. *Boxes*: 25–75% quartiles. *Vertical lines*: range, peak and minimum; *p < 0.05.

Based on the promoter methylation in bladder cancer cell lines, the *ST6GAL1* promoter methylation in primary human bladder cancer samples including CIS (n = 82) was analyzed using MSP technology. NU tissues (n = 23) served as control. All analyzed NU tissues showed unmethylated *ST6GAL1* promoters; representative MSP results demonstrating an aberrant *ST6GAL1* promoter region methylation status in bladder tumors are shown in Figure 4A. Overall, *ST6GAL1* promoter methylation was detected in 32.9% (27/82) of bladder cancer samples. Upon closer examination with respect to the individual growth forms, the methylation frequency in invasive tumors was 53.6% (22/41), whereas only one out of 12 (8.3%) of the CIS samples showed aberrant *ST6GAL1* promoter methylation. The non-invasive pTa phenotype displayed a methylation frequency of 13.8% (4/29). Such disparity between the two growth forms is not surprising, as findings by Wolff et al. suggested a general pattern of hypomethylation in noninvasive urothelial tumors [27].

Subsequently we correlated these methylation results with the *ST6GAL1* mRNA expression in order to determine whether the promoter methylation was responsible for the loss of gene expression in muscle-invasive tumors. Unmethylated NU tissues served as a control (expression level set to 1). Compared to these, unmethylated UC tumors showed a median expression rate of 1.512. In contrast methylated bladder tumors showed a significant (p < 0.05) reduction in *ST6GAL1* mRNA expression down to 0.338 (Figure 4B). The significance of the correlation between loss of *ST6GAL1* gene expression and its promoter methylation was statistically confirmed by using a Fisher’s exact test (p = 0.022) (Table 4).

**Table 4:** **Clinico-pathological parameters in relation to** ***ST6GAL1*** **promoter methylation**

		***ST6GAL1***methylation ^b^				
		***n*** ^***a***^	negative	positive	P-value ^c^	Spearman**rs**
***Parameter:***						
Age at diagnosis						
	≤69 years	29	19	10	0.858	-0.24
	>69 years	31	21	10		
Gender						
	Female	9	6	3	1.000	<0.001
	Male	51	34	17		
Tumor subtype						
	Non-invasive	16	12	4	0.343	0.167
	Invasive	31	18	13		
Histological tumor grade^d^						
	Low grade	14	11	3	0.347	0.139
	High grade	46	29	17		
Tumor stage^d^						
	pTa-pT1	19	15	4	0.073	0.276
	pT2-pT4	27	14	13		
*ST6GAL1* mRNA expression^e^						
	Low	18	9	9	**0.022**	-0.355
	High	30	25	5		

^a^Only patients with primary bladder cancer were included; ^b^based on MSP; ^c^Fisher’s exact test; ^d^according to WHO 2004 classification; ^e^cut-off: 0.5 in relation to NU expression; significant p-values are marked in bold face.

**Figure 5:**
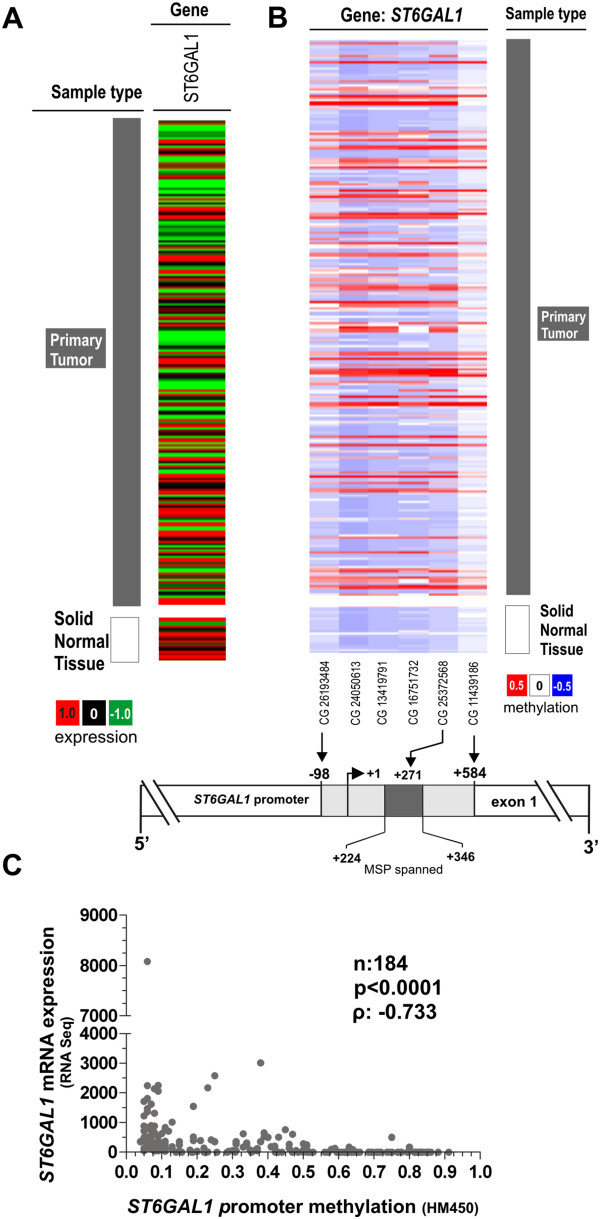
**Tumor-specific** ***ST6GAL1*** **promoter hypermethylation is associated with ST6GAL1 gene silencing in an independent TCGA data set. (A)** *ST6GAL1* expression in bladder tumor samples from the TCGA data portal. *Red*: high expression, black: mean expression and green: low expression. *Left panel*: sample type (dark grey: primary tumor; white: solid normal tissues). *Right panel*: *ST6GAL1* mRNA expression. **(B)** DNA methylation of the *ST6GAL1* promoter analyzed in bladder cancer samples from TCGA data portal. *Red*: high methylation, *white*: mean methylation; *blue*: low methylation. *Right panel*: sample type (dark grey: primary tumor; white: solid normal tissues). *Left panels*: values of *ST6GAL1* DNA methylation for each available CG number. The relative positions of six analyzed CpG duplets within the *ST6GAL1* promoter region covering the MSP analyzed region (+224 to +346) are indicated. +1: *ST6GAL1* TSS. CG25372568 position in relation to the stated TSS: +272. **(C)** Inverse correlation of *ST6GAL1* mRNA expression and its DNA methylation status in primary bladder cancer samples. ρ: *Spearman* correlation coefficient.

In order to strengthen our findings, we analyzed *ST6GAL1* promoter methylation and gene expression in a dataset of independent studies (*The Cancer Genome Atlas* (TCGA) (https://tcga-data.nci.nih.gov)), in total representing 184 different bladder cancer samples (for cohort characteristics see Additional file 1: Table S1). Based on the TCGA data we verified downregulation of *ST6GAL1* gene expression in bladder cancer in comparison to normal bladder tissues (Figure 5A). Furthermore, *ST6GAL1* promoter hypermethylation of six CpG sites (located from -98 bp to +584 bp with respect to the TSS, i.e. covering the MSP analyzed region) was confirmed in bladder cancer samples underscoring a homogenous methylation pattern within the ST6GAL1 promoter locus (Figure 5B) compared to normal solid tissues. Importantly, a highly significant (p < 0.001) inverse correlation (*Spearman* coefficient: -0.733) of aberrant *ST6GAL1* promoter methylation and *ST6GAL1* mRNA expression was verified in this dataset (Figure 5C).

## Discussion

Accumulating evidence shows that ST6GAL1 is aberrantly expressed in various cancer entities such as colon, breast, and epithelial tumor types [14, 17], and most studies propose an oncogenic role for ST6GAL1 [16–20, 28]. However, its role in tumorigenesis remains controversial [21]. Up to date, knowledge about a possible role of ST6GAL1 in human bladder cancer is still lacking. In the present study we aimed to describe for the first time ST6GAL1 expression and regulation in human bladder carcinogenesis.

To begin, we revealed a differential *ST6GAL1* mRNA expression in bladder tumors compared to normal urothelium. Using real-time PCR, we were able to consistently show *ST6GAL1* mRNA expression in normal urothelial tissue. Initial analysis of a mixed-stage tumor cohort failed to reveal a clear pattern of up- or downregulation in *ST6GAL1* expression. However, when these samples were classified according to subtypes/stages, two distinct patterns emerged. Well-differentiated non-invasive papillary tumors were predominately characterized by an increase in ST6GAL1 expression, while invasive tumors (pT2-4) displayed a significant decrease in *ST6GAL1* expression. Such disparity is, however, hardly surprising as both tumor forms are characterized by a unique molecular profile, which extends to the epigenetic level [8, 10, 29]. In addition, Seales et al. showed that expression of oncogenic RAS in HD3 colonocytes caused an increase in α-2,6-sialylation of β1-integrins by ST6GAL1, and the expression of dominant-negative RAS results in decreased sialylation [30]. Therefore, an upregulation of ST6GAL1 in papillary tumors, which often carry a mutated *RAS* gene [7] can be viewed in this context. Thereafter, using patient matched samples, a sequential loss of ST6GAL1 could be demonstrated throughout the course of bladder cancer development beginning in the CIS stage and continuing to invasive disease when compared with the corresponding normal urothelium. Consistent with these findings, we observed weak ST6GAL1 protein expression in invasive bladder tumors. Indeed, more than one fourth of high-grade cancer tissues showed complete loss of ST6GAL1 protein. These observations seem to contradict those by Swindall and colleagues, who reported a general upregulation of ST6GAL1 in epithelial tumors; however, their data was based on a very small sample population of ovarian, stomach and prostate cancers. Most notably, bladder cancer was not included in this cohort [28]. Of clinical importance, in our comprehensive sample collection, ST6GAL1 expression loss correlated with clinico-pathological metastasis, *i.e.* regional lymph node invasion, implying a possible tumor suppressive role for ST6GAL1 in advanced bladder cancer stages.

It is well known that epigenetic alterations, such as DNA methylation, are common mechanisms contributing to tumorigenesis and tumor progression [29, 31, 32]. Analysis of aberrant DNA methylation has rapidly garnered interest in cancer risk assessment, diagnosis, as well as prognosis and therapy monitoring [33, 34]. The progressive increase in *de novo* methylation of CpG islands in urothelial carcinoma suggests that epigenetic gene silencing is involved in the development of UC [10]. Interestingly, Fleischer et al. has previously shown that the *ST6GAL1* promoter sequence contains distinct CpG islands whose DNA methylation caused loss of ST6GAL1 expression in breast cancer [25]. As such, we sought to determine whether promoter methylation could be responsible for *ST6GAL1* gene inactivation in bladder cancer as well. Indeed, we identified a tumor-specific *ST6GAL1* promoter hypermethylation, which appeared to be more frequently associated with poorly differentiated and advanced tumor stages. Importantly, a highly significant inverse correlation between *ST6GAL1* expression and *ST6GAL1* promoter hypermethylation was observed in these bladder tumors. It appears that loss of ST6GAL1 expression in bladder cancer is not only a common event, but also tightly associated with epigenetic changes within the *ST6GAL1* promoter region. This hypothesis was strengthened using an independent bladder tumor data set based on *The Cancer Genome Atlas* (TCGA) portal. Finally, demethylating treatment of methylated *ST6GAL1* bladder cancer cell lines with 5-aza-2-deoxycytidine clearly restored ST6GAL1 expression, thus functionally confirming a strong correlation between *ST6GAL1* expression and its promoter hypermethylation. Our findings give the first indications that this major epigenetic mechanism could be important in the regulation of ST6GAL1 expression in bladder cancer as well. Bearing in mind that this is a known mechanism to mediate the silencing of tumor suppressor genes, a possible tumor suppressive role of ST6GAL1 in the course of bladder cancer progression could be hypothesized even though further studies are needed to analyze, in depth, ST6GAL1 function in human bladder cancer subtypes.

## Conclusions

Our study characterizes for the first time ST6GAL1 expression and regulation during bladder cancer development. ST6GAL1 loss is caused by aberrant *ST6GAL1* promoter methylation potentially indicating a tumor suppressive role in bladder carcinogenesis. Further investigation is warranted in order to more precisely assess the mechanism by which ST6GAL1 contributes to bladder cancer formation, progression and invasion.

## Authors’ information

Pia Antony and Michael Rose are equal first authors.

Nadine T Gaisa and Edgar Dahl are equal co-senior authors.

## Electronic supplementary material

Additional file 1: Table S1: Clinico-pathological parameters of 184 bladder cancer specimens (TCGA) analyzed in this study. (DOC 43 KB)

## Abbreviations

ST6GAL1: Beta-galactosamide alpha-2,6-sialyltransferase 1
mRNA: Messenger ribo nucleic acid
n: Number
NU: Normal urothelium
pTa: Papillary non-invasive tumors
CIS: Carcinoma in situ; pTis
pT2-4: Muscle invasive tumors
PCR: Polymerase chain reaction
TCGA: The Cancer Genome Atlas
HG: High grade
LG: Low grade
IRS: Immunoreactive score
MSP: Methylation specific PCR
DNA: Desoxyribonucleic acid
UC: Urothelial cell cancer
FGFR3: Fibroblast growth factor receptor 3
RAS: Rat sarcoma
TP53: Tumor protein 53
RB: Retinoblastoma
CMP: Cystidine monophosphate
TNFR1: Tumor necrosis factor receptor 1
ATCC: American Type Culture Collection
USA: United States of America
RWTH: Rheinisch Westfälisch Technische Hochschule
EK: Ethics committee
cDNA: Copy number desoxyribonucleic acid
CA: California
WI: Wisconsin
GAPDH: Glyeradehyde 3-phosphate dehydrogenase
MSP: Methylation specific PCR
EDTA: Tris ethylenediaminetetraacetic acid
UV: Ultraviolet
dNTP: Desoxyribonucleosidtriphosphate
DAC: 5-aza-2′-deoxycytidine
TSA: Trichostatin A
FFPE: Formalin fixed paraffin embedded
DAB: 3-3′ diaminobenzidine
FC: Fold change
G1: Well differentiated
G2: Moderately differentiated
G3: Poorly differentiated
WHO: World Health Organization
s.e.m.: Standard error of the margin
PAP: Papillary non-invasive tumors
INV: Invasive tumors
U: Unmethylated
M: Methylated preparation.
