# Supplementary material for: Epigenetic inactivation of ST6GAL1 in human bladder cancer
Source: BMC Cancer. 2014 Dec 2;14:901. doi: 10.1186/1471-2407-14-901 (PMC4265431; doi:10.1186/1471-2407-14-901)
Supplement: Supplementary file 1 — Additional file 1: Table S1: Clinico-pathological parameters of 184 bladder cancer specimens (TCGA) analyzed in this study. (DOC 43 KB) [file 12885_2014_5068_MOESM1_ESM.doc]

| **Additional file 1: Table S1: Clinico-pathological parameters of 184 bladder cancer specimens (TCGA) analyzed in this study** | | | |
| --- | --- | --- | --- |
|
|  | **Categorization** | **n^a^ analyzable** | **%** |
| ***Parameter:*** |  |  |  |
| Gender |  |  |  |
|  | male | 137 | 74.5 |
|  | female | 47 | 25.5 |
| Tumor subtype |  |  |  |
|  | papillary | 55 | 29.9 |
|  | non-papillary | 127 | 69.0 |
|  | unknown | 2 | 1.1 |
| Histological tumor grade^c^ | |  |  |
|  | low grade | 14 | 7.6 |
|  | high grade | 169 | 91.8 |
|  | unknown | 1 | 0.5 |
| Tumor stage^c^ |  |  |  |
|  | pTx | 1 | 0.5 |
|  | pTa | 18 | 9.8 |
|  | pT1 | 1 | 0.5 |
|  | pT2 | 38 | 20.6 |
|  | pT3 | 60 | 32.6 |
|  | pT4 | 27 | 14.7 |
| Lymph node status |  |  |  |
|  | N0 | 112 | 60.9 |
|  | N1 | 17 | 9.2 |
|  | N2 | 33 | 17.9 |
|  | N3 | 6 | 3.3 |
|  | unknown | 16 | 8.7 |
| ^a^Only patients with primary bladder cancer were included; ^b^According to WHO 1973 classification; ^c^According to WHO 2004 classification. | | | |
|
